# MicroRNA-146-5p Promotes Pulmonary Artery Endothelial Cell Proliferation under Hypoxic Conditions through Regulating USP3

**DOI:** 10.1155/2021/3668422

**Published:** 2021-12-07

**Authors:** Wei Zhang, Yujuan Qi, Bo Wu

**Affiliations:** ^1^Department of Cardiac Surgery, Tianjin Chest Hospital, Tianjin, China; ^2^Department of Cardiac Surgery ICU, Tianjin Chest Hospital, Tianjin, China; ^3^Department of Surgery, Tianjin Peace New Century Women and Children's Hospital, Tianjin, China

## Abstract

**Objective:**

MicroRNAs play a pivotal role in the progression of pulmonary hypertension (PAH). Although microRNA-146-5p is specifically expressed in many diseases, but in PAH, its role remains elusive. *Patients and Methods*. 30 patients with PAH and 20 healthy volunteers in our hospital were enrolled, and their serum samples were extracted for the detection of microRNA-146-5p and ubiquitin specific protease 3 (USP3) expression. In addition, the interaction between microRNA-146-5p and USP3 was examined by luciferase reporting assay. Furthermore, the potential mechanism was explored by cell counting kit-8 (CCK-8), 5-ethynyl-2′-deoxyuridine (EdU), and Western blotting experiments.

**Results:**

It was found that microRNA-146-5p was higher in PAH patients than in healthy volunteers. Meanwhile, in hypoxia-induced human pulmonary artery endothelial cell lines (HPAECs), microRNA-146-5p expression was dramatically downregulated while USP3 protein expression was conversely upregulated. Under hypoxic conditions, microRNA-146-5p mimics was able to prompt the growth of HPAECs. In addition, after overexpression of microRNA-146-5p, luciferase reporting assay revealed a reduced luciferase activity of the reporter gene containing the USP3 3′-untranslated region, and a reduction of USP3 protein expression was also confirmed. However, USP3 overexpression partially attenuated the impact of upregulated microRNA-146-5p on the proliferation capacity of HPAECs.

**Conclusions:**

MicroRNA-146-5p was able to enhance the proliferation ability of HPAEC cells under hypoxic conditions through targeting USP3, suggesting the microRNA-146-5p/USP3 axis may act as a target for PAH treatment.

## 1. Introduction

Related diseases caused by pulmonary arterial hypertension (PAH) have gradually become a serious public health problem. Hypoxia may lead to pulmonary vasoconstriction and induce PAH, which may lead to pulmonary vascular reconstruction and the occurrence of pulmonary heart disease [1, 2]. Under the current circumstances, it is of great significance to uncover the mechanism of PAH under hypoxia to improve the prognosis, survival rate, and quality of life of patients with pulmonary heart disease induced by chronic hypoxia pulmonary diseases [3, 4]. Some studies suggested that a variety of growth factors can be produced in the lung during hypoxic conditions to stimulate the proliferation of smooth muscle cells, elastic fibers, and collagen fibers in the inner membrane, resulting in pulmonary vascular remodeling [5, 6]. PAH caused by such vascular remodeling may be relevant to a number of mechanisms such as phenotypic changes of pulmonary smooth muscle cells [7, 8].

Among many clinical molecular markers, microRNA (miRNA), due to its small molecular size and wide role, has gradually attracted wide attention from the medical community in recent years [9, 10]. miRNA is a kind of endogenous noncoding single-stranded RNA encoded by endogenous genes with a length of about 22 nucleotides, which plays a significant role in cell differentiation, proliferation, apoptosis, tumor occurrence, and drug efficiency [11, 12]. Recent studies have also demonstrated that microRNA-146-5p, as a member of miRNA family, is also involved in various stages of cell development, including cell proliferation, apoptosis, and variation [13].

miRNAs can be engaged in the regulation of gene expression and induce protein translation inhibition by partially complementary combining with the 3′ noncoding region of the mRNA of downstream gene, thereby repressing protein synthesis [14, 15]. Bioinformatics analysis demonstrated that microRNA-146-5p could regulate the expression of ubiquitin specific protease 3 (USP3) and bind to it specifically. USP3 is a member of the ubiquitin specific protease (USP) family, which can bind to its target protein through a series of steps by acting on a highly conserved small molecule protein—ubiquitin, mediating the degradation of the target protein or causing other corresponding biological effects [16, 17]. In addition, the research of ubiquitin-specific proteases in diseases is increasingly deepening, including neurodegenerative diseases, hematologic diseases, and infectious diseases, which may serve as a new treatment method for PAH and other diseases in the near future [18, 19].

In this study, we first explored the expression of microRNA-146-5p and USP3 and the interaction between them in pulmonary artery smooth muscle cells under hypoxia conditions, so as to clarify the impacts of microRNA-146-5p and USP3 in the progression and formation of PAH, which may provide a new target for the diagnosis and treatment of PAH.

## 2. Patients and Methods

### 2.1. Clinical Samples

Serum samples from 30 PAH patients aged 36.70 ± 10.31 and 20 healthy volunteers aged 33.02 ± 8.52 were collected and stored in a refrigerator at -80°C. Patients diagnosed with PAH should meet the published guidelines for the diagnosis of pulmonary hypertension: (1) mPAP ≥ 25 mmHg at rest, (2) pulmonary artery wedge pressure ≤ 15 mmHg, and (3) pulmonary vascular resistance (PVR) > 3 Wood units. Patients were excluded if associated with a definite cause, including connective tissue disease, congenital heart disease, chronic pulmonary thromboembolism, and PAH due to left heart disease, lung diseases, and hypoxemia [20–22]. According to the guidelines of the Helsinki Declaration, all subjects in this research signed informed consent, which was approved by the Ethics Committee of Tianjin Chest Hospital. This study was conducted in accordance with the Declaration of Helsinki.

### 2.2. Cell Culture

Human pulmonary artery endothelial cell lines (HPAECs) and human renal epithelial cell line (293T) were purchased from American Type Culture Collection (ATCC) (Manassas, VA, USA), and Dulbecco's modified eagle medium (DMEM) and fetal bovine serum (FBS) were purchased from Life Technologies (Gaithersburg, MD, USA). All cells were cultured with high glucose DMEM containing 10% FBS, penicillin (100 U/mL), and streptomycin (100 *μ*g/mL) in a 37°C, 5% CO_2_ incubator. When grown to 80%-90% confluence, cells were digested with 1x trypsin+EDTA (ethylenediaminetetraacetic acid).

### 2.3. Transfection and Hypoxic Treatment

The control group (miR-NC) and the overexpression vector (microRNA-146-5p mimics) containing the microRNA-146-5p lentiviral sequence were purchased from Shanghai Jima Company (Shanghai, China). Cells were plated in 6-well plates and grown to a cell density of 30-40%, and lentiviral transfection was performed according to the manufacturer's instructions. Cells were collected 48 h later for quantitative real-time polymerase chain reaction (qPCR) and Western blot experiments. In addition, HPAEC cells were exposed to hypoxia (1% O_2_ and 5% CO_2_) at 37°C.

### 2.4. Cell Proliferation Assays

The transfected cells were collected and plated into 96-well plates at 2000 cells per well. After cultured for 24 h, 48 h, 72 h, and 96 h, respectively, 10 *μ*L of cell counting kit-8 (CCK-8) solution (Dojindo, Kumamoto, Japan) was added per well for incubation for 2 hours, and then, the optical density (OD) value of each well was measured in the microplate reader at 490 nm absorption wavelength.

### 2.5. 5-Ethynyl-2′-Deoxyuridine (EdU) Assay

To assess the proliferative capacity of HPAEC cells, EdU proliferation assay (RiboBio, Guangzhou, China) was performed. After transfection for 24 h, the cells were incubated with 50 *μ*m EDU for 2 h, then stained with AdoLo and 4′,6-diamidino-2-phenylindole (DAPI), and the number of EDU-positive cells was examined by fluorescence microscopy. The display rate of EDU positive was shown as the ratio of the number of EDU positive cells to the total DAPI chromogenic cells (blue cells).

### 2.6. Real-Time PCR

Total RNA was extracted from HPAEC cells using TRIzol reagent (Invitrogen, Carlsbad, CA, USA) and reverse transcribed into complementary deoxyribose nucleic acid (cDNA) using Primescript RT Reagent. qPCR reactions were performed using SYBR® Premix Ex TaqTM (TaKaRa, Tokyo, Japan) and StepOne Plus Real-time PCR System. The following primers were used for qPCR reaction: microRNA-146-5p: forward: 5′-GCCCTCTGTGCTACTTACTC-3′, reverse: 5′-GCTGGTTGTGGGTTACTCTC-3′; U6: forward: 5′-GCCCTCTGTGCTACTTACTC-3′, reverse: 5′-GCTGGTTGTGGGTTACTCTC-3′; USP3: forward: 5′-TAGGTATTGTCTACTACTCTG-3′, reverse: 5′-TATATCACTCTTGCTTCA-3′; and *β*-actin: forward: 5′-CCTGGCACCCAGCACAAT-3′, reverse: 5′-TGCCGTAGGTGTCCCTTTG-3′. Data analysis was performed using the ABI Step One software (Applied Biosystems, Foster City, CA, USA), and the relative expression levels of mRNA were calculated using the 2^-*ΔΔ*Ct^ method [23].

### 2.7. Western Blot

The transfected cells were lysed using cell lysis buffer and centrifuged at 14,000 × g for 15 minutes at 4°C. Total protein concentration was calculated by bicinchoninic acid (BCA) Protein Assay Kit (Pierce, Rockford, IL, USA). The extracted proteins were separated using a 10% sodium dodecyl sulphate-polyacrylamide gel electrophoresis (SDS-PAGE) gel and subsequently transferred to a polyvinylidene fluoride (PVDF) membranes (Millipore, Billerica, MA, USA). Western blot analysis was performed according to standard procedures. The primary antibody against USP3 (23303S) and the internal reference GAPDH (5174T) and the secondary antibody were both purchased from Cell Signaling Technology (Danvers, MA, USA).

### 2.8. Dual-Luciferase Reporter Assay

293T cells were seeded in 24-well plates and cotransfected with microRNA-146-5p mimic/NC and pMIR luciferase reporter plasmids. Prior to this, the plasmid was ligated into the pMIR by insertion of other wild-type USP3, paired with the 3′UTR of the USP3 mutation binding site. The plasmid was then introduced into the cells using Lipofectamine 2000 (Thermo Fisher Scientific, Waltham, MA, USA) according to the manufacturer's protocol. After 48 hours of transfection, the reporter luciferase activity was normalized to the control firefly luciferase activity.

### 2.9. Statistical Analysis

The program was processed using the Statistical Product and Service Solutions (SPSS) 22.0 program (IBM, Armonk, NY, USA), and the data were expressed as mean ± standard deviation. *p* < 0.05 was considered to be statistically significant. The continuity variable was analyzed by *t*-test, and the categorical variable was analyzed by *χ*^2^ test or Fisher's exact probability method.

## 3. Results

### 3.1. MicroRNA-146-5p and USP3 Expression in Plasma of Patients with PAH

**Figure 1:**
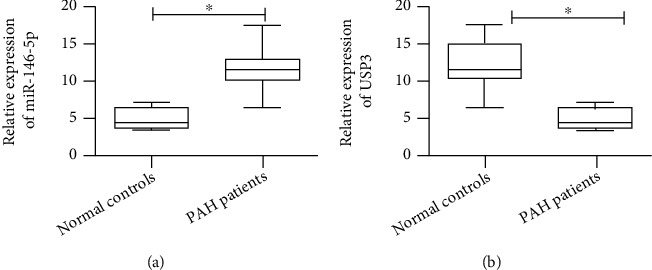
Detection of miR-146-5p and USP3 expression levels in serum from patients with pulmonary hypertension and healthy volunteers. (a) qRT-PCR was used to detect the expression level of miR-146-5p in serum of patients with pulmonary hypertension and healthy volunteers. (b) qRT-PCR was used to detect the expression level of USP3 in serum of patients with pulmonary hypertension and healthy volunteers. The data were mean ± SD, and ^∗^ indicates a significant difference compared with the normal control group (*p* < 0.05).

QPCR detected that PAH patient serum contained higher microRNA-146-5p expression and lower USP3 expression compared to the healthy volunteers (*p* < 0.05; Figures 1(a) and 1(b)).

### 3.2. Hypoxia Decreased MicroRNA-146-5p While Increased USP3 Expression in HPAECs

**Figure 2:**
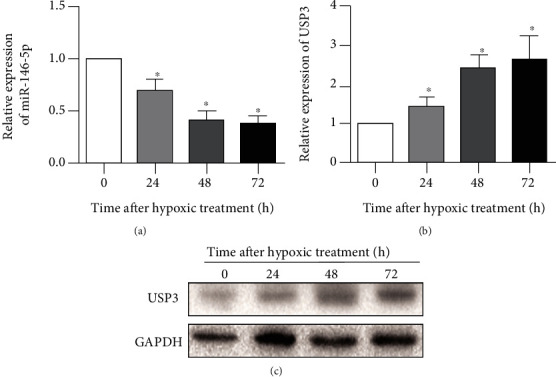
Expression of miR-146-5p and USP3 in HPAECs after hypoxia. (a) qRT-PCR was used to detect the expression levels of miR-146-5p at 0 h, 24 h, 48 h, and 72 h after hypoxia treatment. (b) Western blotting was used to detect the protein expression levels of USP3 at 0 h, 24 h, 48 h, and 72 h after hypoxia treatment. The data were mean ± SD, and ^∗^ indicates a significant difference compared with 0 h of hypoxia (*p* < 0.05).

To determine microRNA-146-5p and USP3 expression in HPAECs under hypoxic conditions, we cultured HPAECs under hypoxic conditions for 0, 24, 48, and 72 h, respectively, and found a dramatic reduction in microRNA-146-5p expression in a time-dependent manner (Figure 2(a)). In addition, USP3 expression was relatively shown as 2.02 ± 0.16, 3.38 ± 0.16, and 3.35 ± 0.42 at 24 hours, 48 hours, and 72 hours, respectively, measured by Western blot assay, suggesting that hypoxia could dramatically induce USP3 protein expression in HPAECs (Figure 2(b)).

### 3.3. MicroRNA-146-5p Upregulation Accelerates Proliferation Rate of HPAECs

**Figure 3:**
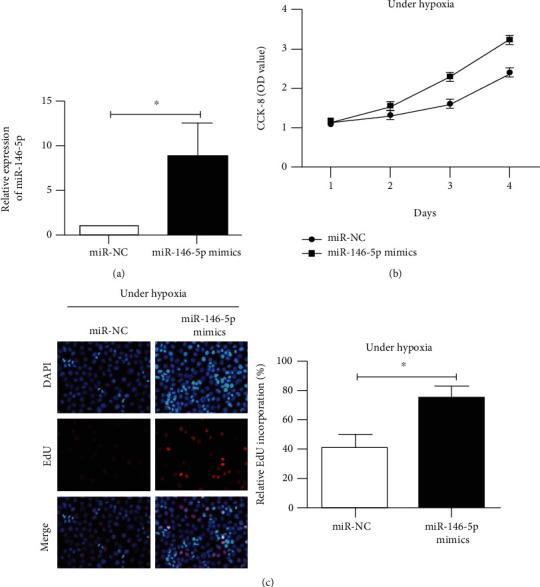
Overexpression of miR-146-5p promotes the proliferative capacity of HPAECs after 48 h of hypoxia. (a) qRT-PCR was used to detect the transfection efficiency after transfection of miR-146-5p overexpression vector after 48 h of hypoxia. (b) CCK-8 detected the proliferative capacity of HPAEC cells after transfection of miR-146-5p overexpression vector after 48 h of hypoxia. (c) The EdU assay detected the number of positive proliferating cells in HPAEC cells after overexpression of miR-146-5p after 48 h of hypoxia. The data were mean ± SD, and ^∗^ indicates a significant difference compared with the miR-NC group (*p* < 0.05).

The microRNA-146-5p overexpression vector was constructed to further evaluate how microRNA-146-5p promotes the growth of HPAECs under hypoxic conditions. After 24 hours of transfection and 48 hours of exposure to hypoxia, qPCR detected an increased microRNA-146-5p level in HPAEC cells induced by overexpression of microRNA-146-5p compared to miR-NC, suggesting a successful construction model (Figure 3(a)). Subsequently, CCK-8 and EdU assays indicated that microRNA-146-5p overexpression dramatically enhanced the proliferation capacity of HPAECs exposed in hypoxia for 48 h compared to the control group (Figures 3(b) and 3(c)).

### 3.4. USP3 Is a Target of MicroRNA-146-5p

**Figure 4:**
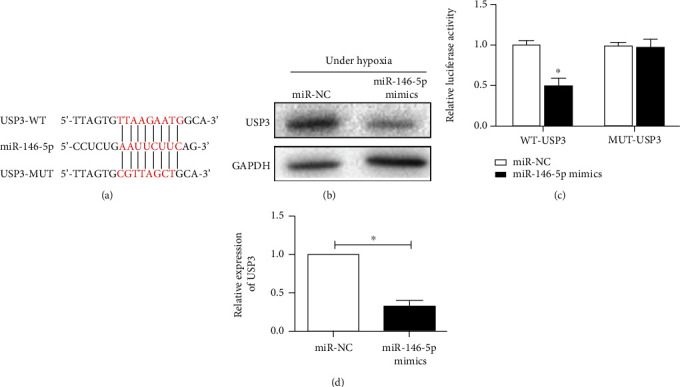
USP3 is a direct binding target gene for miR-146-5p. (a) Schematic representation of miR-146-5p sharing the same complementary seed sequence as the 3′-UTR of USP3. (b) Luciferase reporter assay detected that USP3 is the target gene for miR-146-5p. (c) Western blotting was used to detect the protein expression level of USP3 in HPAEC cells after overexpression of miR-146-5p after 48 h of hypoxia. (d) qRT-PCR was used to detect the mRNA expression level of USP3 in HPAEC cells after overexpression of miR-146-5p after 48 h of hypoxia. The data were mean ± SD, and ^∗^ indicates a significant difference compared with the miR-NC group (*p* < 0.05).

Bioinformatics predicted a potential binding site of microRNA-146-5p in USP3 3′-UTR (Figure 4(a)). And luciferase reporting assay verified that the luciferase activity of 293T cells transfected with WT-USP3-3′UTR vector and microRNA-146-5p overexpression vector was dramatically attenuated after 48 h of hypoxia, while the cytoluciferase activity of cells transfected with MUT-USP3-3′UTR was not affected (Figure 4(b)), which confirmed that microRNA-146-5p could directly target USP3 by binding to its 3′-UTR. In addition, overexpression of microRNA-146-5p reduced USP3 in HPAECs exposed in hypoxia for 48 h at both mRNA and protein levels (Figures 4(c) and 4(d)). The above data suggested that USP3 may act as a target for microRNA-146-5p in HPAECs under hypoxic conditions.

### 3.5. Overexpression of USP3 Partly Alleviates the Impacts of MicroRNA-146-5p on Cell Proliferation Ability of HPAECs under Hypoxia

**Figure 5:**
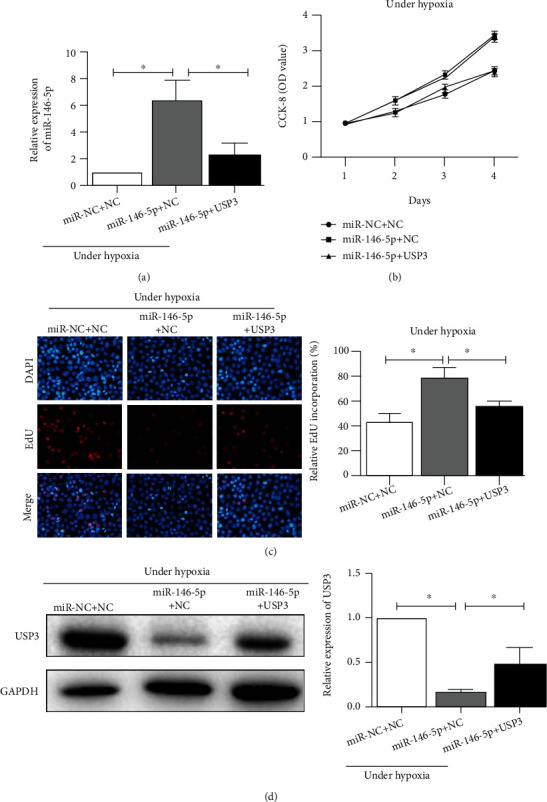
Overexpression of USP3 attenuates the effect of overexpression of miR-146-5p on the proliferative capacity of HPAEC cells after 48 h of hypoxic treatment. (a) qRT-PCR was used to detect the mRNA expression level of miR-146-5p in HPAEC cells after cotransfection of miR-146-5p and USP3 overexpression vectors after 48 h of hypoxia. (b) CCK-8 detected the proliferative capacity of HPAEC cells after cotransfection of miR-146-5p and USP3 overexpression vectors after 48 h of hypoxia. (c) The EdU assay detected the number of positive proliferating cells in HPAECs after cotransfection of miR-146-5p and USP3 overexpression vectors after 48 h of hypoxia. (d) Western blotting was used to detect the protein expression level of USP3 in HPAEC cells after cotransfection of miR-146-5p and USP3 overexpression vectors after 48 h of hypoxia. Data were mean ± SD, and ^∗^ indicates a significant difference (*p* < 0.05).

To further elucidate the regulation of microRNA-146-5p and USP3 on HPAEC cell proliferation under hypoxic conditions, we transfected miR-NC + NC, microRNA-146-5p + NC, or microRNA-146-5p + USP3 into HPAEC cells, followed by hypoxia for 48 hours. qPCR analysis revealed a significant reduction of microRNA-146-5p expression in HPAEC cells transfected with microRNA-146-5p + USP3 compared to those transfected with microRNA-146-5p + NC (Figure 5(a)). In addition, the results of CCK-8 and EdU assays indicated that the proliferation ability of HPAEC cells in the microRNA-146-5p + USP3 group was dramatically weakened compared to those in the microRNA-146-5p + NC group; however, when compared with the miR-NC + NC group, after 48 h hypoxia, the cell proliferation ability was reversed (Figures 5(b) and 5(c)). Additionally, USP3 expression was significantly higher in the microRNA-146-5p + USP3 group than in the microRNA-146-5p + NC group (Figure 5(d)). The above data suggested that USP3 overexpression partially could attenuate the influence of microRNA-146-5p upregulation on cell proliferation capacity under hypoxia.

## 4. Discussion

Pulmonary arterial hypertension (PAH) is a disease typically characterized by pulmonary artery contraction, progressive pulmonary vascular resistance increase, and pulmonary artery pressure rise, which can lead to fatal right heart failure [1–4]. Pulmonary arterioles are composed of endothelial cells, smooth muscle cells, and fibroblasts [5–7]; among which, pulmonary smooth muscle cells are the main biological effector cells in the contraction process of pulmonary arterioles caused by hypoxia and also the key cells involved in the structural reconstruction of pulmonary vessels under the condition of chronic hypoxia [7, 8].

MicroRNA (miRNA), a kind of short single-stranded RNA about 18-25 bp in length, can mediate the physiological and pathological processes of almost all diseases, including cell proliferation, differentiation, migration, carcinogenesis, and apoptosis [9–11]. Previous studies uncover the key role of miRNAs in PAH. Recently, miR-182-3p/Myadm contribute to pulmonary artery hypertension vascular remodeling via a KLF4/p21-dependent mechanism [24]. Besides, miR-483 might reduce experimental pulmonary hypertension by inhibition of multiple adverse responses [25]. In this study, compared with healthy volunteers, patients with PAH had a significant higher expression of microRNA-146-5p but a lower expression of USP3. Previous studies have shown that HPAECs play a crucial role in the process of hypoxia *in vitro*, including promoting cell proliferation and apoptosis, as well as enhancing cell secretion activity. In this investigation, after hypoxia for 24 h, 48 h, and 72 h in HPAEC cells, it was found that the expression of microRNA-146-5p was markedly downregulated while USP3 expression was conversely upregulated, in a time-dependent manner. In HPAEC cells, after overexpression of microRNA-146-5p, CCK-8 and EdU assay revealed an enhanced proliferation ability of HPAEC cells and an elevated positive rate of EdU staining with the increase of time after 48 h of hypoxia culture, suggesting that overexpression of microRNA-146-5p could promote the proliferation activity of pulmonary smooth muscle cells.

miRNA genes are usually located in intron regions, and single strand mature miRNA is produced through a series of processing processes. Mature miRNA molecules form RNA-induced silencing complex (RISC) with Dicer, Argonaute protein, etc. in cells and act on the 3′UTR of specific mRNA to inhibit the translation process or directly degrade the mRNA [11, 12]. For example, microRNA-146-5p could specifically bind to USP3, confirmed in our research by bioinformatics analysis and luciferase reporter gene assay. As a new target gene of microRNA-146-5p, USP3 is found as a member of ubiquitin specific protease (DUBs), which was initially discovered for its cDNA clone fragment had one or two conserved sequences consistent with DUBs. USP3, located on human chromosome 15q22.3, is a functional ubiquitin-specific protease that inhibits ubiquitin-dependent protein degradation [16, 17]. USP can also interact with its target protein through removing the ubiquitin molecules that bind to it, thereby inhibiting the degradation or mediating downstream biological functions of the target protein [18, 19]. In addition, USP3 was dramatically downregulated after the overexpression of microRNA-146-5p. However, under hypoxia conditions, simultaneous overexpression of microRNA-146-5p and USP3 reversed the enhanced proliferation ability induced by microRNA-146-5p. In this study, we firstly uncovered the role of miR-146-5p in PAH by in vitro assay; however, the in vivo assay should be added in the future study.

## 5. Conclusions

In summary, microRNA-146-5p can elevate the proliferation rate of HPAEC cells under hypoxia conditions by targeted modulating USP3, suggesting that microRNA-146-5p/USP3 axis may be a potential target for PAH treatment.

## Data Availability

The datasets used and analyzed during the current study are available from the corresponding author on reasonable request.
